# Disease-Specific Changes in Reelin Protein and mRNA in Neurodegenerative Diseases

**DOI:** 10.3390/cells9051252

**Published:** 2020-05-19

**Authors:** Laia Lidón, Laura Urrea, Franc Llorens, Vanessa Gil, Ignacio Alvarez, Monica Diez-Fairen, Miguel Aguilar, Pau Pastor, Inga Zerr, Daniel Alcolea, Alberto Lleó, Enric Vidal, Rosalina Gavín, Isidre Ferrer, Jose Antonio Del Rio

**Affiliations:** 1 Molecular and Cellular Neurobiotechnology, Institute for Bioengineering of Catalonia (IBEC), Barcelona Institute of Science and Technology (BIST), Science Park of Barcelona, 08028 Barcelona, Spain; llidon@ibecbarcelona.eu (L.L.); lurrea@ibecbarcelona.eu (L.U.); vgil@ibecbarcelona.eu (V.G.); rgavin@ub.edu (R.G.); 2 Department of Cell Biology, Physiology and Immunology, Faculty of Biology, University of Barcelona, 08028 Barcelona, Spain; 3 Center for Networked Biomedical Research on Neurodegenerative Diseases (Ciberned), 28031 Barcelona, Spain; franc.llorens@gmail.com (F.L.); DAlcolea@santpau.cat (D.A.); ALleo@santpau.cat (A.L.); 8082ifa@gmail.com (I.F.); 4 Institute of Neuroscience, University of Barcelona, 08028 Barcelona, Spain; 5 Department of Neurology, Universitätsmedizin Göttingen, 37075 Göttingen, Germany; ingazerr@med.uni-goettingen.de; 6 Bellvitge Biomedical Research Institute (IDIBELL), L’Hospitalet de Llobregat, 08908 Llobregat, Spain; 7 Fundació per a la Recerca Biomèdica i Social Mútua de Terrassa, Terrassa, 08221 Barcelona, Spain; ignacio.alvafer@gmail.com (I.A.); monicadifa@gmail.com (M.D.-F.); miquelaguilar@gmail.com (M.A.); pastorpau@gmail.com (P.P.); 8 Memory Disorders Unit, Department of Neurology, Hospital Universitari Mutua de Terrassa, Terrassa, 08221 Barcelona, Spain; 9 German Center for Neurodegenerative Diseases, 37075 Göttingen, Germany; 10 Memory Unit, Department of Neurology, Institut d’Investigacions Biomèdiques Sant Pau—Hospital de Sant Pau, Universitat Autònoma de Barcelona, 08041 Barcelona, Spain; 11 Centre de Recerca en Sanitat Animal (CReSA, IRTA-UAB), Campus de la Universitat Autònoma de Barcelona, Bellaterra, 08193 Catalonia, Spain; enric.vidal@irta.cat; 12 Department of Pathology and Experimental Therapeutics, University of Barcelona, 08907 Barcelona, Spain; 13 Senior Consultant, Bellvitge University Hospital, Hospitalet de Llobregat, 08907 Barcelona, Spain; 14 Bellvitge Biomedical Research Institute (IDIBELL), Hospitalet de Llobregat, 08908 Barcelona, Spain

**Keywords:** Reelin, Creutzfeldt-Jakob disease, Alzheimer’s disease, Parkinson’s disease dementia, a-synucleopathies, cerebrospinal fluid

## Abstract

Reelin is an extracellular glycoprotein that modulates neuronal function and synaptic plasticity in the adult brain. Decreased levels of Reelin activity have been postulated as a key factor during neurodegeneration in Alzheimer’s disease (AD) and in aging. Thus, changes in levels of full-length Reelin and Reelin fragments have been revealed in cerebrospinal fluid (CSF) and in post-mortem brains samples of AD patients with respect to non-AD patients. However, conflicting studies have reported decreased or unchanged levels of full-length Reelin in AD patients compared to control (nND) cases in post-mortem brains and CSF samples. In addition, a compelling analysis of Reelin levels in neurodegenerative diseases other than AD is missing. In this study, we analyzed brain levels of *RELN* mRNA and Reelin protein in post-mortem frontal cortex samples from different sporadic AD stages, Parkinson’s disease with dementia (PDD), and Creutzfeldt-Jakob disease (sCJD), obtained from five different Biobanks. In addition, we measured Reelin protein levels in CSF samples of patients with mild cognitive impairment (MCI), dementia, or sCJD diagnosis and a group of neurologically healthy cases. The results indicate an increase in *RELN* mRNA in the frontal cortex of advanced stages of AD and in sCJD(I) compared to controls. This was not observed in PDD and early AD stages. However, Reelin protein levels in frontal cortex samples were unchanged between nND and advanced AD stages and PDD. Nevertheless, they decreased in the CSF of patients with dementia in comparison to those not suffering with dementia and patients with MCI. With respect to sCJD, there was a tendency to increase in brain samples in comparison to nND and to decrease in the CSF with respect to nND. In conclusion, Reelin levels in CSF cannot be considered as a diagnostic biomarker for AD or PDD. However, we feel that the CSF Reelin changes observed between MCI, patients with dementia, and sCJD might be helpful in generating a biomarker signature in prodromal studies of unidentified dementia and sCJD.

## 1. Introduction 

The extracellular glycoprotein Reelin plays relevant roles during development, circuit maturation, and synapse maintenance of the central nervous system (CNS) [1,2,3]. The full-length Reelin protein (≈420 kD) is cleaved by several extracellular proteases. Three different cleavages have been reported: (i) the N-t cleavage (between 1244 and 1245 aa), mediated by metalloproteinases, such as a disintegrin and metalloproteinases with thrombospondin motifs (ADAMTS) family, (ii) WC cleavage (between 3455 and 3456 aa) mediated by proprotein convertase family proteases, and (iii) C-t cleavage (between 2688 and 2689 aa) mediated by serine proteases such as tissue plasminogen activator or ADAMTS-4 and ADAMTS-5 or meprin metalloproteases [4,5,6,7,8]. Thus, full-length Reelin (≈420 kD) generates smaller N-term proteolyzed peptides of ≈310 and ≈180 kD, together with a ≈190 kD central domain peptide and additional lower molecular weight C-term peptides [4,5,6,7,8]. The levels of Reelin in biological samples are usually determined by analyzing the presence of either the full-length (≈420 kD) or the ≈180–190 kD band in Western Blots using N-term directed antibodies (i.e., G10 or 142). However, with some controversy [5,8,9,10], it seems that the N-t cleavage of Reelin is able to inactivate Reelin function by ADAMTS 3, at least during embryonic development [11].

It is believed that Reelin levels in adult brain must be maintained to a certain level to ensure neural homeostasis since its decrease may lead to synaptic dysfunction and neurodegeneration. Indeed, the level changes of Reelin in biological fluids (i.e., cerebrospinal fluid (CSF)) have been analyzed by several groups during neurodegeneration (typically Alzheimer’s disease (AD)) and aging. These studies report either decreased [12] or unchanged levels [13,14,15,16,17,18,19] of full-length Reelin or fragments between AD compared to control cases. In addition, results published analyzing the levels of ≈180–190 kD also show differences. Furthermore, very few data have been published describing putative Reelin changes in the CSF in other neurodegenerative diseases (i.e., α-synucleiopathies). A correlative study of the brain levels and CSF levels of Reelin in different neurodegenerative diseases is lacking. In a previous publication our group [20] developed a study to analyze the changes in *RELN* mRNA and protein levels in sporadic Creutzfeldt-Jakob disease (sCJD) postmortem samples [20]. In the present study, we expand on this to explore in detail the putative changes of full-length Reelin and *RELN* levels in post-mortem samples of neocortex and Reelin protein levels in CSF samples. We analyzed the *RELN* and Reelin levels in brain samples of AD(III-IV) and AD(V-VI), Parkinson’s disease with dementia (PDD), and sCJD cases compared to non-degenerative (nND) samples. In addition, we analyzed through Western Blotting the Reelin protein levels in CSF samples obtained from patients with mild cognitive impairment (MCI), dementia, and sCJD compared with control cases. 

The results indicate an increase in *RELN* mRNA in frontal cortex (area 8) from nND to AD(V-VI) stages and in sCJD, in contrast to PDD and early AD(III-IV). However, Reelin protein levels in post-mortem frontal cortex samples were unchanged between nND and AD(V-VI) or PDD. For CSF, Reelin levels decreased in the CSF of dementia cases compared to controls and MCI patients. These Reelin changes correlate with observed levels of amyloid β-protein and pTau in the CSF of dementia and control cases. With respect to sCJD, there was a tendency to increase in brain samples compared to nND and to decrease in CSF with respect to controls.

## 2. Materials and Methods

### 2.1. Human Samples

**Figure 1:**
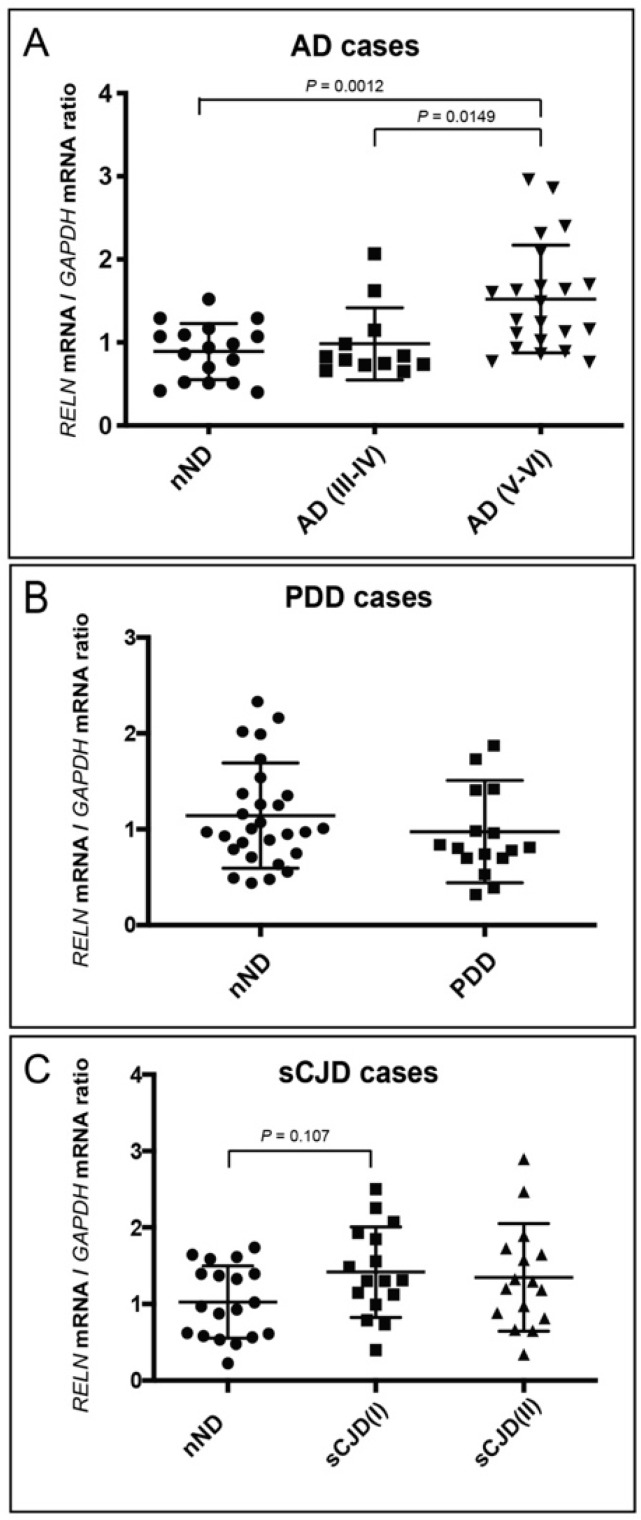
*RELN* mRNA quantification in postmortem brain samples by RT-qPCR in AD (**A**), PDD (**B**), and sCJD (type I and II) (**C**) compared with non-degenerative (nND) samples using *GAPDH* for standardization. Each dot corresponds to one postmortem sample. The values were calculated from the *RELN* mRNA value and normalized to *GAPDH* value from the same cDNA preparation; the mean ± S.D. for each group of samples is displayed. Only *p* values indicating statistical differences between groups are displayed in the graphs (except in (**C**)). *p* values were determined using ANOVA (Bonferroni post hoc test).

The brains of nND and patients with sCJD, PDD, or AD were obtained from 3 to 8 h after death and were immediately prepared for morphological and biochemical studies. A total of 246 frontal cortex (area 8) post-mortem samples and CSF were obtained from the Hospital Clinic de Barcelona, HUB-ICO-IDIBELL Biobank, Hospital de Sant Pau (SPIN Cohort [21], Hospital Universitario Mutua de Terrassa, and the UMG (Universitätsmedizin Göttingen, Germany). In order to avoid biobank-associated differences between samples, the samples were distributed in a blind basis between Germany and Spain laboratories. In practical terms, some frozen tissue (FT) was processed in Germany and Spain. Thus, FT from AD, PDD, sCJD and nND from different biobanks were distributed between Germany and Spain. Indeed, in some cases the same FT sample was half divided and processed for RT-qPCR and Western Blot. RT-qPCR of AD and sCJD brain samples was performed in Germany and the RT-qPCR of PDD samples was performed in Spain using the same protocols (see below). In each case, blind nND samples from the nND pool of the Table S1 was processed in parallel to patient data. The Western Blotting determination of AD and PDD brain samples with blind nND samples (obtained from the pool) was developed in Spain. The numbers of samples plotted in each condition were as follows: nND (n = 41), AD(III-VI) (n = 55, 12 (III-IV) and 43 (V-VI)), PDD (n = 40), and sCJD (n = 36). In Table S1, we provided the main data (age, gender, etc.) concerning the plotted cases in all figures. We described each sample as FT (frozen tissue used for qPCR or Western Blot) or CSF (for Western Blot). As a fragment of the FT was used for Western Blotting and the rest for mRNA extraction, some FT patient samples were shared for qPCR and Western Blotting. In the particular case of the nND, this was the situation. In the case of the AD (III-IV), 12 samples were used for qPCR in Figure 1. Concerning AD(V-VI) a total of 43 samples were used: 43 for Western Blot (Figure 4) and, of these, 22 for qPCR, randomly selected (Figure 1). For sCJD(I), a total of 20 samples were used. Of these, we blindly selected 16 for qPCR and 16 for Western Blot (Figure 4). In the case of sCJD(II), a total of 16 samples were used for qPCR (Figure 1) and 10 were selected for Western Blotting (Figure 4), all selected blindly. Concerning PDD, a total of 40 samples were used. Of these, 28 were blindly selected for Western Blot (Figure 4) and 16 for qPCR (Figure 1). Thus, as some samples were used in parallel in different laboratories for RT-qPCR or Western Blotting experiments, we plotted for each figure the number of samples used in each determination as well as the mean ± standard deviation (S.D.).

In addition, we collected CSF that was neurologically healthy (n = 40), with mild cognitive impairment (MCI) (n = 28), dementia (DEM) (n = 44), sCJD (n = 20), and PDD (n = 11), and processed the samples for Western Blotting as indicated [22] (Figure 5). For biosafety reasons, CSF from sCJD patients was recruited between 2017 and 2019 following the protocol described in [23] and together with nND CSF samples processed at CReSA (IRTA-UAB, Barcelona). The rest of the CSF samples were collected between 2014 and 2018 in Spain (see Table S1). In all cases, subjects underwent a standard neurological exam, a Mini Mental State Examination (MMSE), functional and behavioral evaluation, and, when possible, a complete neuropsychological assessment. Diagnosis of MCI, dementia, and PDD was made according to the recommended criteria [24,25,26]. All sCJD cases were classified according to the previous diagnostic consensus criteria [27]. For CSF of DEM, MCI and control levels for human p-Tau, total Tau, and amyloid β-protein were measured using ELISA kits (INNOTEST^TM^ Phospo-Tau181p, INNOTEST^TM^ hTau Ag and INNOTEST^TM^ β-amyloid (1–42); all from Innogenetics, Belgium) following the manufacturer’s instructions; for Dementia cases, advanced diagnostic research criteria as well as internal CSF-PET validation was used to improve AD diagnosis [28,29]. Written informed consent was obtained from participant subjects or their relatives. The present study was approved by the ethics committees of the collaborating groups.

For biochemical studies of postmortem brain cases, samples of frontal cortex were frozen in liquid nitrogen and stored at –80 °C until use. For routine neuropathological diagnosis, 4% formalin-fixed, formic acid-treated samples were embedded in paraffin. The neuropathological study was carried out on de-waxed 4-μm-thick paraffin sections of the frontal, primary motor, primary sensory, parietal, temporal superior, temporal inferior, anterior gyrus cinguli, anterior insular, and primary and associative visual cortices; entorhinal cortex and hippocampus; caudate putamen and globus pallidus; medial and posterior thalamus; hypothalamus; Meynert nucleus; amygdala; midbrain (two levels), pons, and bulb; and cerebellar cortex and dentate nucleus. The sections were stained with hematoxylin and eosin, Klüver Barrera, and, for immunohistochemistry, to a panel of different antibodies. All the antibody details used in the characterization as well as the following sections can be seen in Table S2.

### 2.2. Immunohistochemical Methods

The immunohistochemical detection of Reelin in post-mortem tissue is a challenging issue [30]. In our study, a total of 16 post-mortem tissue samples (not included in Table S1) of nND (6), PDD (3), and AD (7) were used. Of these, 11 (nND and AD) were samples from the hippocampal region and 12 (4 nND, 4 AD and 3 PDD) from frontal cortex area 8. All samples were obtained from the HUB-ICO-IDIBELL Biobank. At autopsy, samples from the frontal cortex and hippocampal formation were fixed with buffered 4% paraformaldehyde for 24 h, cryoprotected with 30% sucrose, frozen on dry ice, and stored at –80 °C until use. Following neuropathological examination, Sections (40 to 50 μm thick) were obtained in a freezing microtome (Leica, Germany) and processed. For immunohistochemistry, the sections were processed as follows: after rinsing in phosphate buffered-saline (PBS: KH_2_PO_4_ 1.058 mM; NaCl 155.17 mM; Na_2_HPO_4_-7H_2_O 2.96 mM) endogenous peroxidase, activity was inhibited by a solution of 10% methanol and 3% H_2_O_2_ for 25 to 30 min. After blocking in a solution containing 10% normal serum for 3 h, free-floating sections were incubated overnight with mouse monoclonal α−Reelin antibody (clone 142, Table S2) at 4 °C. All primary antibodies were diluted in PBS containing 5% normal serum, 0.2% gelatin, and 0.5% Triton X-100. Tissue-bound primary antibody was detected utilizing the ABC method (Vector Laboratories, UK). Peroxidase activity was revealed using 0.03% DAB and 0.01% hydrogen peroxide. Afterwards, sections were mounted onto gelatinized slides and air-dried, dehydrated in graded alcohols, cleared in xylene, and coverslipped with Eukitt (Merck, Germany). In addition, each experiment included a control section processed without the primary antibodies, and this always resulted in the absence of immunostaining. Immunoreacted sections were analyzed in an Olympus BX61. Photomicrographs were obtained using a DP72 12.5 Megapixel cooled digital color camera. Pictures were only modified in brightness and contrast using Fiji^TM^, and figures were designed using GraphPad Prism^TM^ 8.3.0 software (GPS-1625480-L###).

### 2.3. Western Blotting and Quantification

Samples from different sources were processed for Western Blotting. In each gel, randomly selected nND and patient-samples were included on a blind basis. For each pathology, Reelin level was compared with blind-selected nND samples loaded in the same gels. The collected brain samples were homogenized in (10% *w/v*) 50 mM Tris-HCl, pH 7.4/150 mM NaCl/0.5% Triton X-100/0.5% Nonidet P-40, containing protease and phosphatase inhibitors (Protease and Phosphatase Inhibitor Cocktail, Sigma Aldrich, Dorset, UK; Cat. Number PPC1010, final concentration of 1%(*v/v*) in the homogenization buffer). After this, samples were centrifuged at 15,000 *g* for 20 min at 4 °C. The resulting supernatant was normalized for protein content using the bicinchoninic acid assay (BCA) protein assay kit (Pierce, Cat. number 23225). For CSF, the protein contents were determined and normalized using the BCA kit. Sample (brain and CSF) extracts were boiled at 96 °C for 3 min [22,31], and 15 μg of total were loaded in each gel well followed by 5%–6% SDS electrophoresis. Afterwards, they were transferred to nitrocellulose membranes for 2 h at 4 °C. For CSF, following transfer, the nitrocellulose membrane was stained with Ponceau S (Sigma Aldrich) and then scanned (see below) (Figure S1) previous to the Reelin detection. Membranes were then blocked with 5% not-fat milk in Tris-buffered saline (Tris-HCl 20 mM/ NaCl 150 mM; pH. 7.4) for 2 h and incubated overnight in 0.5% blocking solution containing primary antibodies. After incubation with peroxidase-tagged secondary antibodies (1:2000 diluted), membranes were revealed by an ECL-plus chemiluminescence Western Blot kit (Amershan-Pharmacia Biotech, Piscataway, NJ, USA). In our experiments, each nitrocellulose membrane from post-mortem samples was used to detect Reelin (G10 or 142) and tubulin (brain samples), or Reelin alone (CSF cases). 

For quantification, Ponceau-stained and antibody-incubated and developed films were scanned at 2400 × 2400 dpi (i800 MICROTEK high quality negative film scanner), and the densitometric analysis was made using Quantity One^TM^ Image Software Analysis (s/n 540BR, Biorad, Hercules, CA, USA). Reelin protein levels derived from brain samples were correlated with tubulin levels. For CSF, Reelin levels were corrected to the ≈50 kD Ponceau band. Thus, for each sample, the densitometric levels of Reelin and the reference protein ladder (tubulin or Ponceau band) was obtained from the same nitrocellulose membrane avoiding misleading data. All plotted data are represented as the mean ± standard deviation (S.D.). The normality of the data distributions was checked via the Shapiro–Wilk test. Statistical analysis was analyzed using ANOVA with follow-up Bonferroni post hoc tests used to delineate all pairwise comparisons following a significant effect. In all cases, statistical analysis was developed using GraphPad Prism^TM^ 8.3.0 software (GPS-1625480-L###). As indicated, data in plots are presented as the mean ± S.D. Differences between groups were considered as tendency at *p* > 0.1 and statistically significant at * *p* < 0.05 and ** *p* < 0.01.

### 2.4. Real-Time Quantitative Polimerase Chain Reaction (RT-qPCR)

Quantitative real time PCR was performed on total RNA extracted with a mirVana’s isolation kit (Ambion, Austin, TX, USA) from the frontal cortex of human samples. Purified RNAs were used to generate the corresponding cDNAs, which served as PCR templates for mRNA quantification. PCR amplification and detection were performed with the ROCHE LightCycler 480 detector, using 2× SYBR GREEN Master Mix (Roche, Basel, Switzerland) as reagent, following the manufacturer’s instructions. The reaction profile was: denaturation–activation cycle (95 °C for 10 min) followed by 40 cycles of denaturation–annealing–extension (95 °C for 10 min, 72 °C for 1 min, 98 °C continuous). mRNA levels were calculated using the Light Cycler 480 software (Roche). Data were analyzed with SDS v. 1.9.1 Software (Applied Biosystems, Madrid, Spain) following the 2^−*ΔΔ*CT^ method of Applied Biosystems. Primers were as follows: *RELN* (5’-actctgtcaacagctcaagc-3’) and (3’-tggtcaattgcccagctttg-5). The results were normalized for the expression levels of the housekeeping gene, *GAPDH* (5’-tccaaaatcaagtggggcga -3’ and 3’-tctccatggtggtgaagacg -5’), which were quantified simultaneously with the target gene in each sample.

### 2.5. Selection Criteria for Reelin Analysis in CSF Samples and RT-qPCR

As indicated, analysis of CSF Reelin levels in AD yielded diverse results (see Introduction). Thus, as CSF Reelin levels could be affected by sample storage and handling conditions [15,22,31], we first checked the quality of the obtained mRNA after processing as well as the pattern band in Western Blot. Samples with an RNA integrity number (RIN) lower than 7 [32] as well as CSF samples without a clear resolution after the electrophoresis and the blotting of the two highest molecular weight bands of Reelin (≈420 + 310 bands) and the lower bands were rejected for quantification (see Figure S1 as example). These problems were first observed in [19], showing the absence of the upper bands or Reelin in the CSF samples used in their study. As indicated, in our study, a total of 143 CSF samples were analyzed. Of these, 26 nND, 20 MCI, 29 DEM, 9 sCJD, and 11 PDD were measured, analyzed, and plotted in the Figure 5; overall, then, 66.43 % of the samples were considered for quantification. Details of gender, age, disease, and biobank origin of processed samples are included in Table S1.

## 3. Results

First, we aimed to determine putative *RELN* changes in postmortem brain extracts by quantitative PCR in the different neurodegenerative diseases compared to nND samples (Figure 1). For AD cases, the RT-qPCR quantification of *RELN* showed an increase in AD(V-VI) (1.523 ± 0.649, mean ± S.D.) compared to nND (0.89 ± 0.339, mean ± S.D.; ** *p* = 0.00128) and AD(V-VI) compared to AD(III-IV) (0.984 ± 0.435, mean ± S.D.; * *p* = 0.0149) (Figure 1A). No changes in *RELN* were observed between nND and PDD samples (Figure 1B). A tendency for *RELN* to increase, albeit non-significant, was also observed (*p* = 0.107) in sCJD(I) (1.417 ± 0.590, mean ± S.D.) and to a lesser extend (*p* > 0.1) compared to sCJD(II) (1.349 ± 0.7034, mean ± S.D) in comparison to their respective nND (1.026 ± 0.471, mean ± S.D.) (Figure 1C). 

Next, we analyzed the Reelin pattern of staining in the hippocampal formation of AD(V-VI) and the frontal cortex of AD(V-VI) and PDD compared to nND. The immunohistological detection of Reelin using currently available antibodies in post-mortem tissue is a challenging issue, and few studies have shown positive, consistent results (i.e., [12,33,34]). 

**Figure 2:**
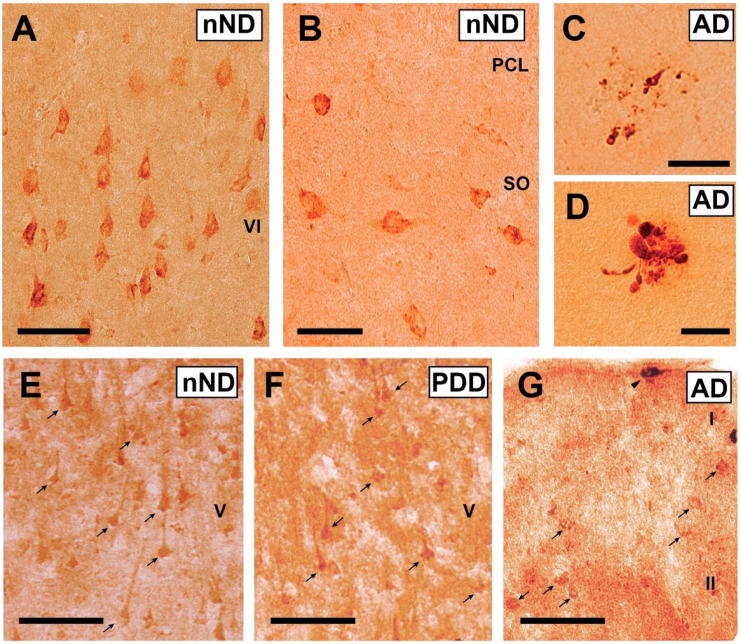
Examples of Reelin-immunostained cells in postmortem human samples. (**A**) Pyramidal neurons of layer VI of temporal cortex of an nND case. (**B**) Non-pyramidal neurons immunoreactive for Reelin in the stratum oriens (SO) of an nND case. (**C**,**D**) Examples of groups of Reelin-positive dystrophic neurites in the hippocampus proper (stratum oriens) in two different AD cases. (**E**,**F**) Photomicrograph illustrating Reelin-positive pyramidal-shaped neurons (arrows) in isocortical layer V in nND and PDD cases. (**G**) Example of Reelin positive cells in layers I-II (arrows) of the frontal cortex of an AD case. Notice the presence of a horizontally oriented Cajal-Retzius cell (arrowhead) in layer I. Abbreviations: PCL: Pyramidal cell layer. Scale bars: A–B = 75 μm; C–D = 75 μm; E–F = 100 μm; G = 100 μm.

We used the antibody against Reelin (clone 142) in frozen tissue (Figure 2) following the protocol described by [35]. In the present study, the distribution of Reelin in *corpora amylacea* [14] was not analyzed. After documentation, we were able to see that Reelin was expressed by both pyramidal shaped neurons in the hippocampal formation and frontal cortex and non-pyramidal cells (mainly located in upper isocortical layers) (Figure 2). In the hippocampus, the pyramidal cell layer of the CA2-CA3 was labeled as well as the subiculum (Figure 2A). In addition, Reelin was localized in non-pyramidal neurons with different shapes in the plexiform layers of the hippocampus (Figure 2B). As observed in other studies, we were able to detect groups of Reelin-positive dystrophic neurites in the hippocampal formation (Figure 2C), but only in AD(V-VI) cases. These aggregates were not observed in the frontal cortex of AD cases or in PDD samples due to the relevant background that impaired putative cell quantification.

**Figure 3:**
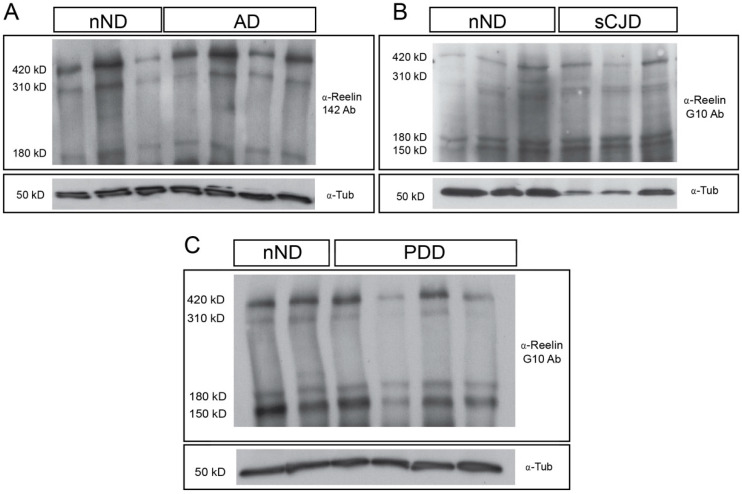
Examples of Western Blotting determination of Reelin in post-mortem brain samples of representative neurodegenerative diseases AD (**A**), sCJD (**B**), and PDD (**C**) with respect to nND. As observed, each quantified gel contains both nND and patient samples. Each gel well was loaded with 15 μg of protein. The four-band labelling using 142 (A) or G10 (**B**,**C**) antibodies can be seen. Reelin-probed membranes were immunoblotted using antibodies against Tubulin for standardization.

**Figure 4:**
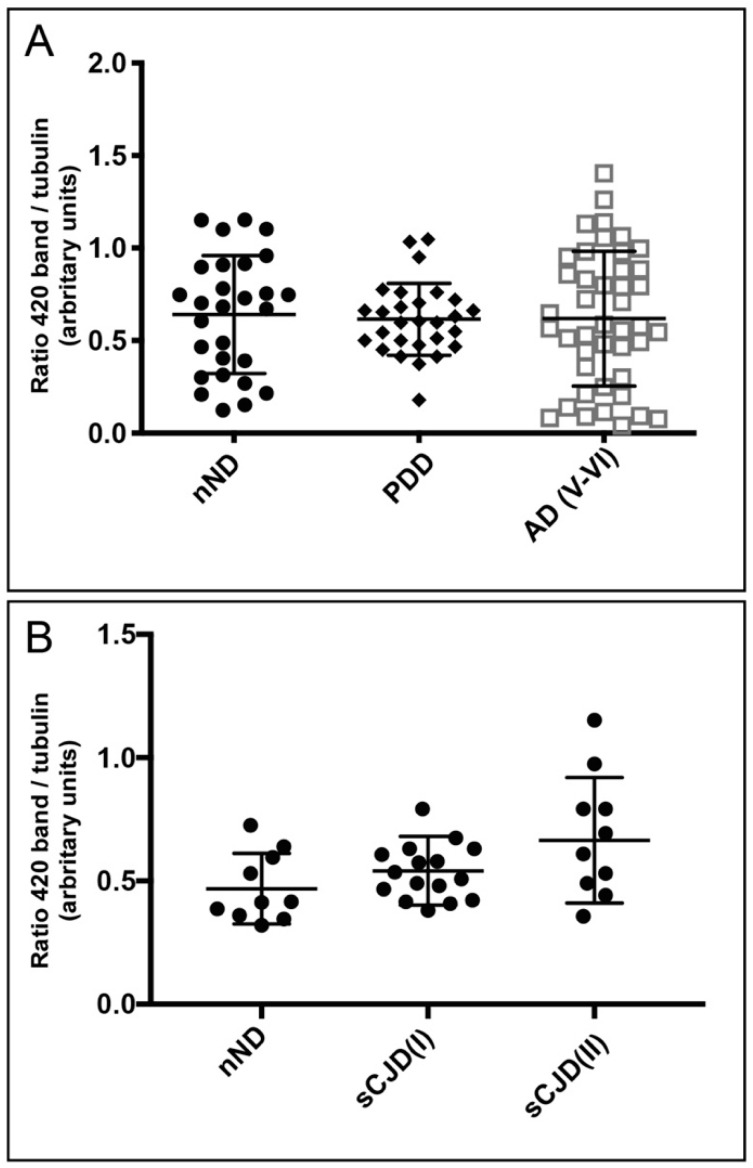
Plots illustrating the densitometric quantification of Reelin-revealed films of different neurodegenerative diseases. PDD and AD(V-VI) (**A**) and sCJD (I-II) (**B**) with respect to nND. Each dot corresponds to one sample, and the mean ± S.D. for each group is also displayed.

Next, we sought to corroborate *RELN* changes using the Western Blotting detection of Reelin (Figure 3 and Figure 4). Brain extracts from the frontal cortex were immunoblotted using the N-terminal-directed antibodies G10 or 142. The two antibodies render a similar band pattern in the different diseases analyzed (Figure 3). Four relevant bands may be defined in brain extracts: ≈420 (full-length Reelin), ≈310, ≈180, and ≈150 kD, in addition to a smear at low molecular weight (Figure 3, Figure S2). However, only the upper two bands can be clearly distinguished in CSF samples, which were clearly identified, and an intense band ≈150–180 kD was observed in revealed films (Figure S3). 

The densitometric analysis of the ≈420 kD Reelin band in developed films determined non-significant differences in levels of Reelin in AD(V-VI) and PDD compared to nND post-mortem brain samples (Figure 4A) (i.e., AD(V-VI): 0.6184 ± 0.363, mean ± S.D. and PDD: 0.6155 ± 0.194, mean ± S.D., compared to nND: 0.640 ± 0.318, mean ± S.D.). With respect to sCJD, a moderate non-statistically significant increase in Reelin was found in sCJD (I and II) compared to nND (i.e., sCJD(I): 0.541 ± 0.139, mean ± S.D.; and sCJD(II) 0.6647 ± 0.2543, mean ± S.D. compared to nND: 0.4683 ± 0.143, mean ± S.D.) (Figure 4B). 

**Figure 5:**
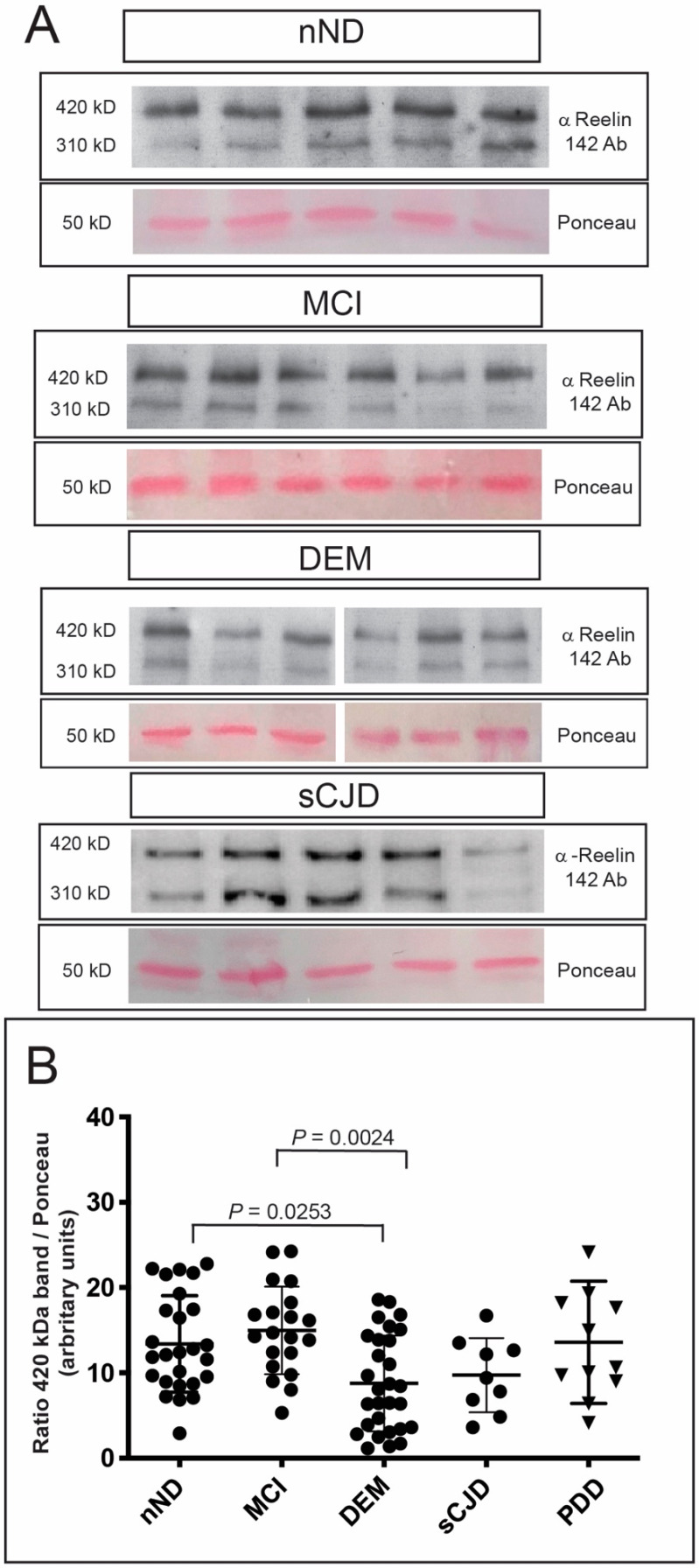
(**A**) Examples of the Western Blotting determination of Reelin in the CSF of different patients (MCI, DEM, sCJD) and their controls (nND). By way of example, the upper bands of ≈420 and ≈310 kD of the revealed films of nND, MCI, DEM, and sCJD are shown. For each gel the Ponceau staining is also shown. (**B**) Graph illustrating the results of the densitometric measurement of the ≈420 kD band in the different CSF samples. Each dot corresponds to one sample, and the mean ± S.D. for each disease and nND is also displayed. Only *p* values indicating statistical differences between groups are displayed in the graphs. *p* values were determined using ANOVA (Bonferroni post hoc test).

Lastly, Reelin levels were checked in selected CSF samples (see Material and Methods, Figure 5). We checked the ≈420 kD band density (Figure 5A) corrected for the ≈50 KD band in Ponceau S-stained nitrocellulose (see Figure S1 as example). Densitometric analysis showed decreased ≈420 kD Reelin levels in DEM compared to nND (DEM: 8.778 ± 5.617, mean ± S.D. and nND: 13.39 ± 5.647, mean ± S.D.; * *p* = 0.0253), although a tendency to decreased levels was also observed in sCJD cases compared to nND cases (from 9.734 ± 4.340 (sCJD) to 13.39 ± 5.647 (nND), mean ± S.D.; *p* = 0.7286) (Figure 5B). In addition, a statistical decrease was also observed between MCI compared to DEM cases (from 14.980 ± 5.126 (MCI), mean ± S.D. to 8.778 ± 5.617 (DEM); ** *p* = 0.0024) (Figure 5B). This last result suggests an opposing tendency between increased *RELN* synthesis in brain parenchyma and decreasing ≈420 kD Reelin levels in CSF of MCI and DEM compared to nND. In contrast, no significant differences were found between PDD compared to nND (13.58 ± 7.156 (PDD) mean ± S.D. to 13.39 ± 5.647 (nND), mean ± S.D. (Figure 5B). 

A summary of the obtained results can be seen in Table 1.

## 4. Discussion

Several reports support a protective role of Reelin in preventing cognitive decline in AD and tauopathy mouse models [36,37,38]. Although the role of Reelin in adult processes such us cell proliferation and maturation is not fully clear [36,39], its active role in preventing amyloid β-protein-mediated synaptic dysfunction is well established [36,37]. Although not described for α-synuclein, in vitro levels of Reelin can be increased by stress-mediating factors such us β-sheet enriched aggregated peptides (i.e., amyloid β-protein or prion-mimicking peptides) [15]. However, overproduced Reelin under these conditions, leading to increased reactive oxygen species in treated cells, is unable to trigger Disabled-1 (Dab1)-mediated intracellular signaling in target neurons [15]. As indicated above, full-length Reelin can be proteolyzed by several kinases (see Introduction). Although with some discrepancies [5,8,10], it seems that the N-t cleavage decreases Dab1 phosphorylation mediated by Reelin [8,10,11]. Although these experiments have been developed in embryonic mice or in vitro, they open the question of whether only measuring the amount of the ≈180 kD proteolyzed band of Reelin is a clear measure of Reelin activity. For this reason, we preferred to analyze total levels of Reelin and their mRNA in the different diseases.

Concerning the several neurodegenerative diseases analyzed in the present study, we found a progressive increase in *RELN* mRNA from nND or AD (III-IV) to AD(V-VI) stages in frontal cortex (area 8), which suggests increased *RELN* transcription in parallel with advanced neurodegenerative AD stages. The *RELN* changes are in line with some published studies [33,40] but in contrast with [12]. Concerning full-length Reelin (≈420 kD) protein levels in post-mortem brain extracts, no differences were observed between nND or AD(III-IV) (not shown) and AD(V-VI) samples in contrast to [41]. In addition, in contrast to [22], we found a significant reduction in ≈420 kD full-length Reelin between control and DEM cases in CSF samples from two different Biobanks (HSP and HMT). Furthermore, full-length Reelin levels decreased between MCI and DEM stages, and no differences were observed between nND and MCI. This decrease in Reelin in the CSF of DEM patients was also recently noted in the data published by Dayon and coworkers [42], and it occurs as well in other diseases such as schizophrenia (i.e., [43]). In fact, several publications by the same group have reported varying results for full-length Reelin levels in CSF (i.e, non-significant increase in [17] or non-significant decrease in [22]). Our decrease in full-length Reelin in DEM CSF samples correlates with lower amyloid β-protein = 608.63 ± 172.76 pg/mL (mean ± S.D.) but higher pTau = 97.46 ± 40.95 pg/mL (mean ± S.D.) in contrast to nND, with higher Reelin content and amyloid β-protein = 960.63 ± 402.1 pg/mL (mean ± S.D.) and lower pTau = 43.66 ± 24.63 pg/mL (mean ± S.D.) content in CSF samples. This in turn correlates with: (i) classical studies indicating that the lack of either Reelin, Dab1, or both VLDLR and ApoER2 exhibits hyperphosphorylation of tau [44,45], and those that indicate that increased Reelin decreased pTau levels, and (ii) studies indicating the a reduction in Reelin expression results in enhanced amyloidogenic APP processing, as indicated by the precocious production of amyloid-β peptides, and a significant increase in the number and size of amyloid-β plaques in brain parenchyma. In addition, levels of amyloid β-protein and pTau in MCI samples in our study were slightly different from those observed in Dementia cases: amyloid β-protein = 665.63 ± 366.21 pg/mL (mean ± S.D.) and pTau = 84.01 ± 34.21 pg/mL (mean ± S.D.). However, and surprisingly, levels of Reelin in CSF from patients with MCI (including patients with vascular involvement or psychiatric disorders) without classical AD level markers in the CSF were similar to those observed in MCI with AD markers or nND, with or without little amyloid plaque formation. These results could be related to the fact that amyloid β-protein is able to bind Reelin in aggregates, leading to a parallel decrease in both proteins in CSF. However, we cannot rule out a different regulation of *RELN* promoter between MCI with AD markers in CSF compared to MCI without AD markers. Taking into account that *RELN* promoter regulation depends on various factors (see [40] for details), further studies are needed to determine the specific modulation of *RELN* promoter in different neurodegenerative diseases (i.e., those affecting neocortical compared to hippocampal regions, or displaying MCI in the presence or absence of AD markers in CSF). For example, hypermethylation levels of the 5′ region of *RELN* promoter, leading to lower Reelin production, have been described, although with some discrepancies, in autism [46], temporal lobe epilepsy [47], and schizophrenia [48,49]. However, a recent study reported no changes in the methylation of 5′ region of the *RELN* promoter [40] compared to nAD cases, although DNA-methyltransferase 1 (the main enzyme responsible for the hypomethylation of *RELN* promoter) is downregulated in AD brains and *RELN* mRNA levels are higher than nND in the study. This suggests that other mechanisms could play a role in controlling *RELN* promoter activity and Reelin production. However, we cannot rule out an area-specific epigenetic modulation of the *RELN* promoter (i.e., archicortex compared to isocortex) that might be differentially modulated during neurodegenerative progression (especially in AD).

In contrast, no differences in *RELN* expression and full-length Reelin protein levels were observed in either brain sample or CSF from frontal cortex between nND/controls and PDD patients, in agreement with what was reported by Botella-López et al. [22] for CSF samples. To our knowledge, this study is the first to illustrate unchanged levels of Reelin in the neocortex of PDD patients. PDD samples used in the present study were categorized as Braak stages 5–6 and derived from different biobanks. Histologically, these samples showed phosphorylated α-synuclein in histological sections mainly in upper isocortical layers as well as relevant staining in axonal tracts. However, additional studies are needed to ascertain why *RELN* and full Reelin protein levels are not modified in the frontal cortex and the CSF of PDD patients.

With respect to sCJD, our data indicate a tendency to increased *RELN* and ≈420 kD Reelin expression in sCJD, reinforcing our previously published data [20] In addition, we used samples (brain samples and CSF) from two different biobanks: HUB-ICO-IDIBELL in and UMG in the present study, with similar results. Since *RELN* mRNA and protein levels in infective prion mouse models might occur only at the final stages of the disease, we believe that this change reflects the profound metabolic changes strongly present in the final stages of sCJD [20]. In addition, Reelin generated under these conditions is not functional, as it is in AD [18], which might enhance cognitive decline and disease progression. 

However, full-length Reelin levels in CSF decreased in DEM and sCJD. In this respect, we might hypothesize the sequestration of Reelin in extracellular deposits of amyloid β-protein or pathogenic PrP, with lower amounts able to reach the CSF (see also [50]), in parallel to PrP or amyloid β-protein levels in CSF of sCJD patients [51,52,53] and DEM (i.e., [54]) respectively.

In the adult rodent and ferret, Reelin-positive cells in the isocortex are GABAergic, mainly in Calbindin, Calretinin, NPY-, and Somatostatin, but only rarely in Parvalbumin-, Cholecystokinin-, or VIP-positive interneurons [33,55,56,57]. In humans, as described by Impagnatiello et al., Reelin expression in non-pyramidal neurons in the prefrontal cortex is mainly in the upper isocortical layers [58,59]. However, Roberts et al. described Reelin immunoreactivity in pyramidal and non-pyramidal neurons in the isocortex [60]. This dual expression of Reelin in the isocortical regions was corroborated in monkey brains [35]. In our study, the relevant labeling of non-pyramidal neurons with different morphologies was observed in upper layers I-II of the frontal cortex while more diffuse labelling was observed in pyramidal neurons in other layers, reinforcing these previous descriptions in human [60] and monkey [35] isocortex. 

In parallel, numerous studies have reported high levels of *RELN* expression in particular populations of the hippocampal formation and parahippocampal cortex: layer II pyramidal projecting cells of the entorhinal cortex, pyramidal neurons of the subiculum, and CA3-CA2 and GABAergic neurons located in lower layers of the entorhinal cortex and the stratum-lacunosum moleculare, and molecular layer of the fascia dentata of the hippocampus [30,33,61]. In fact, the colocalization of Reelin and amyloid β-protein has been demonstrated in layer II pyramidal neurons of the entorhinal region in AD post-mortem brains [30]. In addition to this, other subcortical regions also present relevant Reelin staining (i.e., see [35,62] for examples). With respect to hippocampal formation, Reelin colocalizes with amyloid β-protein plaques in AD and rodent models of AD (i.e., [63,64,65]). In our study, we uncovered the presence of aggregates of Reelin-positive dystrophic neurites in human hippocampal formation of post-mortem AD samples. This Reelin/amyloid β-protein interaction correlates with memory impairments observed during AD evolution and in normal aging [33,64]. In fact, it seems that in AD mouse models, layer II pyramidal cells co-expressing Reelin in the entorhinal cortex are more sensitive to amyloid β-protein accumulation, leading to their cell death [30], which reduces *RELN* expression and transport to the hippocampus [33], thereby enhancing amyloid plaque formation [66] and cognitive deficits. 

In isocortex, where pyramidal and GABAergic neurons express Reelin, it has been found that relevant numbers of Calbindin-positive cells decrease in AD human patients [67] but not in PDD [68]. In our study, we corroborated the immunohistochemical expression pattern of Reelin, but we also determined biochemically that there was a progressive increase in *RELN* during the temporal evolution of AD. However, full-length Reelin levels were unchanged in brain parenchyma but decreased in CSF in DEM stages of AD. The decrease in GABAergic neurons (particularly Calbindin-positive cells of layer I-II, including Cajal-Retzius cells of layer I) might affect Reelin levels in susceptible patients with increased levels of *RELN* mRNA. However, as indicated, extracellular Reelin might accumulate in amyloid β-protein containing plaques [63,65] or in corpora amylacea [14]. Thus, taking into account our observations and these previous descriptions, we envision a scenario resembling a “degenerating loop” during AD progression, based on: (i) the progressive presence of different amyloid β-protein species during AD evolution, leading to (ii) cell death of subsets of Reelin-positive and -negative cells, and (iii) Reelin accumulation in amyloid plaques or corpora amylacea in the entorhinal, hippocampal, and other subcortical structures [62], and then returning again to the first step in this “degenerative loop”. This increased amyloid β-protein delivery potentiates the expression of *RELN* mRNA in neighboring cells (see [40] for a recent study) but also enhances inflammation and reactive stress, which in turn also potentiates the generation of a non-functional *RELN* expression unable to overcome the progressive decline of cognitive function. Thus, as a result of this accumulation, there are unchanged or non-significantly different levels of full-length Reelin observed in most studies. 

Nevertheless, our data indicating increased *RELN* mRNA levels corroborate previously published studies (see Introduction for references) and could explain the divergent data from studies regarding Reelin levels in CSF. Additionally, we must not forget that, in all our studies, values for Reelin measured by Western Blots (brain extracts or CSF) were normalized, with control proteins determined in each sample in two ways: by measuring the total amount of protein with BCA, and with reference proteins/bands in the same membrane (i.e., tubulin or Ponceau S staining of reference bands). Unfortunately, this has not been explored in some published studies, which might contribute to the differing results reported by some groups (see above mentioned references). However, in our study, we rejected 33.56 % of the CSF samples. Furthermore, available Reelin ELISA Kits are useful for plasma-derived samples (i.e., [69,70]) but not in CSF or postmortem brains. In addition, a recent study demonstrates that Reelin levels displayed significant intraindividual variation in the course of 24 h in the plasma in healthy individuals [71]. As previously noted, the plasma level of Reelin are largely conditioned by liver production and show relevant changes in some liver-associated diseases [72,73,74,75]. During the course of our study, we tried to use some commercial ELISA kits for Reelin determination in CSF, without achieving consistent or reliable results. Thus, the development of techniques complementary to the Western Blotting determination of Reelin content will help researchers. However, we expected an advance in the development of CSF kits for Reelin as is the case for other proteins (i.e., pTau, amyloid β-protein). Due to its particular processing and maintenance, first indicated by [19], Reelin levels in CSF cannot be considered a diagnostic biomarker for AD or PDD. However, we feel that the CSF Reelin changes observed between MCI and DEM in CSF and sCJD might be helpful in generating a biomarker signature in prodromal studies of AD and sCJD.

## Figures and Tables

**Table 1:** Table summarizing *RELN* and Reelin protein changes in the postmortem frontal cortex (FC) observed in the present study between the different neurodegenerative diseases (ND) analyzed or in CSF samples after diagnosis (DIAG; i.e., MCI or DEM) (see Material and Methods for details) compared to specific nND cases. Symbols: ↑ = statistical increase; ↓: statistical decrease; >: tendency (non-statistically significant; *p* ≈ 0.1) to increase; <: tendency (non-statistically significant, *p* ≈ 0.1) to decrease; -: no changes. The *RELN* increase between AD(V-VI) compared to AD(III-IV) in FC samples is plotted using the ⇑ symbol. In addition, the Reelin decrease between DEM compared to MCI in CSF samples is also plotted using the ⇓ symbol.

**ND**	***RELN* in FC**	**Reelin in FC**
AD(III-IV)	-	-
AD(V-VI)	↑, ⇑	-
PDD	-	-
sCJD(I)	>	>
sCJD(II)	-	>
**DIAG**	**Reelin in CSF**	
MCI	-	
DEM	↓, ⇓	
PDD	-	
sCJD	<	

## Supplementary Materials

The following are available online at https://www.mdpi.com/2073-4409/9/5/1252/s1, Figure S1. Examples of the Western Blotting determination of Reelin in the CSF of different samples to illustrate the CSF rejection criteria. Figure S2: Example of Western Blot determination of Reelin in the post-mortem brain samples of nND and AD using the 142 Ab. Figure S3: Example of Western Blot determination of Reelin in the CSF samples. Table S1: List of the quantified cases in the study. Table S2: List of the antibodies used in this study.
