# Supplementary material for: Disease-Specific Changes in Reelin Protein and mRNA in Neurodegenerative Diseases
Source: Cells. 2020 May 19;9(5):1252. doi: 10.3390/cells9051252 (PMC7290479; doi:10.3390/cells9051252)
Supplement: Supplementary file 1 [file cells-09-01252-s001.zip › Table S1.pdf]

Table S1 . List of the quantified cases in the study

Control (nND) cases

Gender	Age	Biobank	Sample
Male	58	HC	FT
Male	76	HC	FT
Male	62	HC	FT
Female	74	HC	FT
Male	70	HC	FT
Female	81	HC	FT
Male	78	HC	FT
Male	83	HC	FT
Male	78	HC	FT
Male	64	HC	FT
Male	86	HC	FT
Female	90	HC	FT
Female	88	HC	FT
Female	73	HC	FT
Female	83	HC	FT
Female	74	HC	FT
Female	86	HC	FT
Male	69	HUB-ICO-IDIBELL	FT
Male	64	HUB-ICO-IDIBELL	FT
Male	67	HUB-ICO-IDIBELL	FT
Male	74	HUB-ICO-IDIBELL	FT
Male	86	HUB-ICO-IDIBELL	FT
Female	73	HUB-ICO-IDIBELL	FT
Male	70	HUB-ICO-IDIBELL	FT
Male	85	HUB-ICO-IDIBELL	FT
Male	62	HUB-ICO-IDIBELL	FT
Male	59	HUB-ICO-IDIBELL	FT
Male	39	HUB-ICO-IDIBELL	FT
Male	78	HUB-ICO-IDIBELL	FT
Male	61	HUB-ICO-IDIBELL	FT
Male	84	HUB-ICO-IDIBELL	FT
Male	72	HUB-ICO-IDIBELL	FT
Male	65	HUB-ICO-IDIBELL	FT
Male	25	HUB-ICO-IDIBELL	FT
Male	43	HUB-ICO-IDIBELL	FT
Male	59	HUB-ICO-IDIBELL	FT
Male	56	HUB-ICO-IDIBELL	FT
Male	43	HUB-ICO-IDIBELL	FT
Male	56	HUB-ICO-IDIBELL	FT
Female	65	HUB-ICO-IDIBELL	FT
Female	66	HUB-ICO-IDIBELL	FT
Male	68	HSP	CSF

Female	65	HSP	CSF
Female	55	HSP	CSF
Male	61	HSP	CSF
Female	59	HSP	CSF
Female	68	HSP	CSF
Male	67	HSP	CSF
Female	56	HSP	CSF
Male	59	HSP	CSF
Male	56	HSP	CSF
Female	58	HSP	CSF
Female	56	HSP	CSF
Male	58	HSP	CSF
Male	65	HSP	CSF
Female	61	HSP	CSF
Male	64	HSP	CSF
Male	63	HSP	CSF
Male	62	HSP	CSF
Female	71	HSP	CSF
Male	70	HSP	CSF
Male	70	UMG	CSF
Male	22	UMG	CSF
Female	66	UMG	CSF
Female	38	UMG	CSF
Male	56	UMG	CSF
Female	47	UMG	CSF

Mild cognitive impairement (MCI) with AD markers cases

Gender	Age	Biobank	Sample
Female	71	HMT	CSF
Male	74	HMT	CSF
Male	65	HMT	CSF
Male	75	HMT	CSF
Female	87	HMT	CSF
Female	71	HMT	CSF
Female	64	HMT	CSF
Female	74	HMT	CSF
Female	70	HMT	CSF
Female	57	HMT	CSF
Male	64	HMT	CSF
Female	81	HMT	CSF
Male	65	HMT	CSF
Male	73	HMT	CSF
Female	75	HMT	CSF
Male	84	HMT	CSF
Female	85	HMT	CSF
Male	69	HMT	CSF
Female	77	HMT	CSF
Male	67	HMT	CSF

AD Dementia cases

Gender	Age	Biobank	Sample
Female	70	HMT	CSF
Male	77	HMT	CSF
Female	61	HMT	CSF
Female	78	HMT	CSF
Male	66	HMT	CSF
Male	79	HMT	CSF
Male	72	HMT	CSF
Female	81	HMT	CSF
Male	72	HMT	CSF
Male	52	HMT	CSF
Male	71	HMT	CSF
Female	53	HMT	CSF
Female	72	HMT	CSF
Male	81	HMT	CSF
Female	65	HMT	CSF
Male	85	HMT	CSF
Male	69	HMT	CSF
Female	69	HMT	CSF
Female	62	HMT	CSF
Female	55	HMT	CSF
Male	79	HMT	CSF
Female	81	HMT	CSF
Male	58	HMT	CSF
Male	69	HSP	CSF
Male	63	HSP	CSF
Male	82	HSP	CSF
Male	60	HSP	CSF
Male	57	HSP	CSF
Male	69	HSP	CSF

AD cases (III-IV)

Gender	Age	Biobank	Sample
Female	67	HUB-ICO-IDIBELL	FT
Female	71	HUB-ICO-IDIBELL	FT
Male	69	HUB-ICO-IDIBELL	FT
Male	64	HUB-ICO-IDIBELL	FT
Female	90	HUB-ICO-IDIBELL	FT
Male	83	HUB-ICO-IDIBELL	FT
Female	81	HUB-ICO-IDIBELL	FT
Female	77	HUB-ICO-IDIBELL	FT
Female	84	HUB-ICO-IDIBELL	FT
Female	84	HUB-ICO-IDIBELL	FT
Male	84	HUB-ICO-IDIBELL	FT
Male	79	HUB-ICO-IDIBELL	FT

AD cases (V-VI)

Gender	Age	Biobank	Sample
Male	75	HC	FT
Male	90	HC	FT
Female	77	HC	FT
Female	87	HC	FT
Male	85	HC	FT
Female	81	HC	FT
Female	85	HC	FT
Female	86	HC	FT
Female	89	HC	FT
Male	79	HC	FT
Male	81	HC	FT
Female	73	HC	FT
Male	92	HC	FT
Male	88	HC	FT
Female	93	HC	FT
Female	75	HC	FT
Male	78	HC	FT
Female	81	HC	FT
Male	77	HC	FT
Female	90	HC	FT
Female	88	HC	FT
Female	93	HC	FT
Male	88	HC	FT
Female	77	HC	FT
Male	83	HC	FT
Male	92	HC	FT
Female	83	HC	FT
Female	90	HC	FT
Male	87	HC	FT
Female	82	HC	FT
Female	85	HC	FT
Female	92	HC	FT
Male	81	HC	FT
Female	81	HC	FT
Female	80	HC	FT
Female	88	HC	FT
Female	88	HC	FT
Male	79	HC	FT
Female	90	HC	FT
Female	87	HC	FT
Female	90	HC	FT
Female	86	HC	FT
Male	85	HC	FT
Female	82	HUB-ICO-IDIBELL	FT
Female	56	HUB-ICO-IDIBELL	FT
Male	87	HUB-ICO-IDIBELL	FT

Female	75	HUB-ICO-IDIBELL	FT
Male	87	HUB-ICO-IDIBELL	FT
Male	75	HUB-ICO-IDIBELL	FT
Male	82	HUB-ICO-IDIBELL	FT
Male	77	HUB-ICO-IDIBELL	FT
Female	96	HUB-ICO-IDIBELL	FT
Female	67	HUB-ICO-IDIBELL	FT

<u>PDD cases</u>

Gender	Age	Biobank	Sample
Female	69	HC	FT
Male	81	HC	FT
Female	70	HC	FT
Male	76	HC	FT
Female	77	HC	FT
Female	70	HC	FT
Female	79	HC	FT
Male	71	HC	FT
Female	87	HC	FT
Male	68	HC	FT
Female	73	HC	FT
Female	77	HC	FT
Male	72	HC	FT
Female	81	HC	FT
Male	73	HC	FT
Female	87	HC	FT
Female	71	HC	FT
Male	74	HC	FT
Male	77	HC	FT
Female	77	HC	FT
Male	80	HC	FT
Male	77	HC	FT
Female	88	HC	FT
Female	84	HC	FT
Female	81	HC	FT
Male	68	HC	FT
Male	82	HC	FT
Male	80	HC	FT
Female	69	HC	FT
Male	81	HC	FT
Female	87	HC	FT
Male	74	HC	FT
Male	78	HC	FT
Male	71	HC	FT
Male	81	HC	FT
Female	83	HC	FT
Female	90	HC	FT
Male	62	HC	FT

Female	81	HC	FT
Male	83	HC	FT
Female	73	HMT	CSF
Male	65	HMT	CSF
Male	73	HMT	CSF
Female	72	HMT	CSF
Female	80	HMT	CSF
Male	55	HMT	CSF
Male	74	HMT	CSF
Male	74	HMT	CSF
Female	71	HMT	CSF
Female	70	HMT	CSF
Male	65	HMT	CSF

<u>sCJD cases</u>

Gender	Age	PrP Type	Biobank	Sample
Female	61	ND	UMG	CSF
Male	76	ND	UMG	CSF
Female	73	ND	UMG	CSF
Female	76	ND	UMG	CSF
Male	59	ND	UMG	CSF
Male	66	ND	UMG	CSF
Female	71	ND	UMG	CSF
Female	66	ND	UMG	CSF
Male	72	ND	UMG	CSF
Male	25	1	HUB-ICO-IDIBELL	FT
Male	79	1	HUB-ICO-IDIBELL	FT
Female	82	1	HUB-ICO-IDIBELL	FT
Female	63	1	HUB-ICO-IDIBELL	FT
Female	54	1	HUB-ICO-IDIBELL	FT
Female	65	1	HUB-ICO-IDIBELL	FT
Male	52	1	HUB-ICO-IDIBELL	FT
Male	61	1	HUB-ICO-IDIBELL	FT
Female	60	1	HUB-ICO-IDIBELL	FT
Male	56	1	HUB-ICO-IDIBELL	FT
Male	74	1	HUB-ICO-IDIBELL	FT
Female	87	1	HUB-ICO-IDIBELL	FT
Female	85	1	HUB-ICO-IDIBELL	FT
Female	70	1	HUB-ICO-IDIBELL	FT
Male	55	1	HUB-ICO-IDIBELL	FT
Male	73	2	HUB-ICO-IDIBELL	FT
Female	67	2	HUB-ICO-IDIBELL	FT
Female	63	2	HUB-ICO-IDIBELL	FT
Female	76	2	HUB-ICO-IDIBELL	FT
Female	52	2	HUB-ICO-IDIBELL	FT
Male	76	2	HUB-ICO-IDIBELL	FT
Female	77	2	HUB-ICO-IDIBELL	FT
Male	5	2	HUB-ICO-IDIBELL	FT

Female	61	2	HUB-ICO-IDIBELL	FT
Male	73	2	HUB-ICO-IDIBELL	FT
Female	76	2	HUB-ICO-IDIBELL	FT
Female	72	1	HUB-ICO-IDIBELL	FT
Male	46	1	HUB-ICO-IDIBELL	FT
Male	64	1	HUB-ICO-IDIBELL	FT
Male	65	1	HUB-ICO-IDIBELL	FT
Male	70	1	HUB-ICO-IDIBELL	FT
Male	66	2	HUB-ICO-IDIBELL	FT
Female	76	2	HUB-ICO-IDIBELL	FT
Male	65	2	HUB-ICO-IDIBELL	FT
Female	47	2	HUB-ICO-IDIBELL	FT
Male	54	2	HUB-ICO-IDIBELL	FT

Abbreviations.

CSF = Cerebrospinal fluid for protein analysis
ND = Non determined
FT = Frozen Tissue for protein and RNA analysis
HC = Hospital Clinic
HMT = Hospital Mutua de Terrassa
HSP = Hospital de Santa Creu i Sant Pau
HUB-ICO-IDIBELL = Hospital de Bellvitge and the Oncology Institute
UMG = Universitätsmedizin Göttingen
