# Supplementary material for: Disease-Specific Changes in Reelin Protein and mRNA in Neurodegenerative Diseases
Source: Cells. 2020 May 19;9(5):1252. doi: 10.3390/cells9051252 (PMC7290479; doi:10.3390/cells9051252)
Supplement: Supplementary file 1 [file cells-09-01252-s001.zip › Table S2.pdf]

Table S2: Characteristics of the antibodies used.

Antibody	Mono-/polyclonal	Dilution	Supplier	Country
AT8 (Ser202/Thr205)	Monoclonal	1:500	Innogenetics	Ghent, BE
β-Amyloid	Monoclonal	1:50	Boerhinger	Germany
CD68	Monoclonal	1:100	Dako	Glostrup, DK
GFAP	Rabbit polyclonal	1:250	Dako	Glostrup, DK
Ubiquitin	Rabbit polyclonal	1:200	Dako	Glostrup, DK
PrP (3F4)	Monoclonal	1:1000	Dako	Glostrup, DK
αB-crystallin	Monoclonal	1:100	Adcam	Cambridge, USA
α-Synuclein	Rabbit polyclonal	1:500	Millipore	Billerica, USA
α-Reelin (142)	Monoclonal	1:500	Chemicon	Billerica, USA
α-Reelin (G19)	Monoclonal	1:500	Chemicon	Billerica, USA
Tubulin	Monoclonal	1:5000	Sigma	UK
