# Supplementary figures and images for: Disease-Specific Changes in Reelin Protein and mRNA in Neurodegenerative Diseases
Source: Cells. 2020 May 19;9(5):1252. doi: 10.3390/cells9051252 (PMC7290479; doi:10.3390/cells9051252)

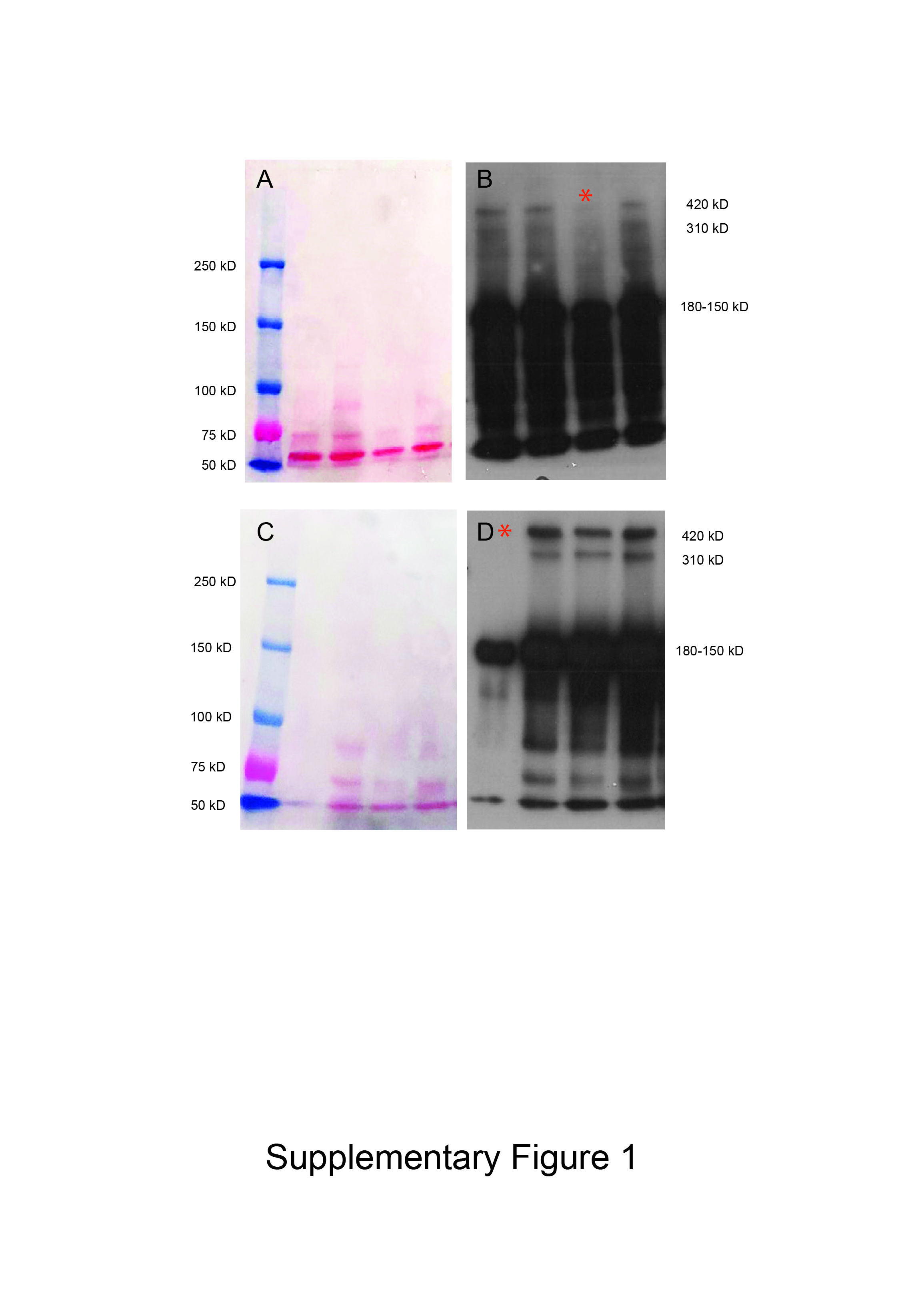

Supplement: Supplementary file 1 [file cells-09-01252-s001.zip › Supplementary Fig 1.jpg]

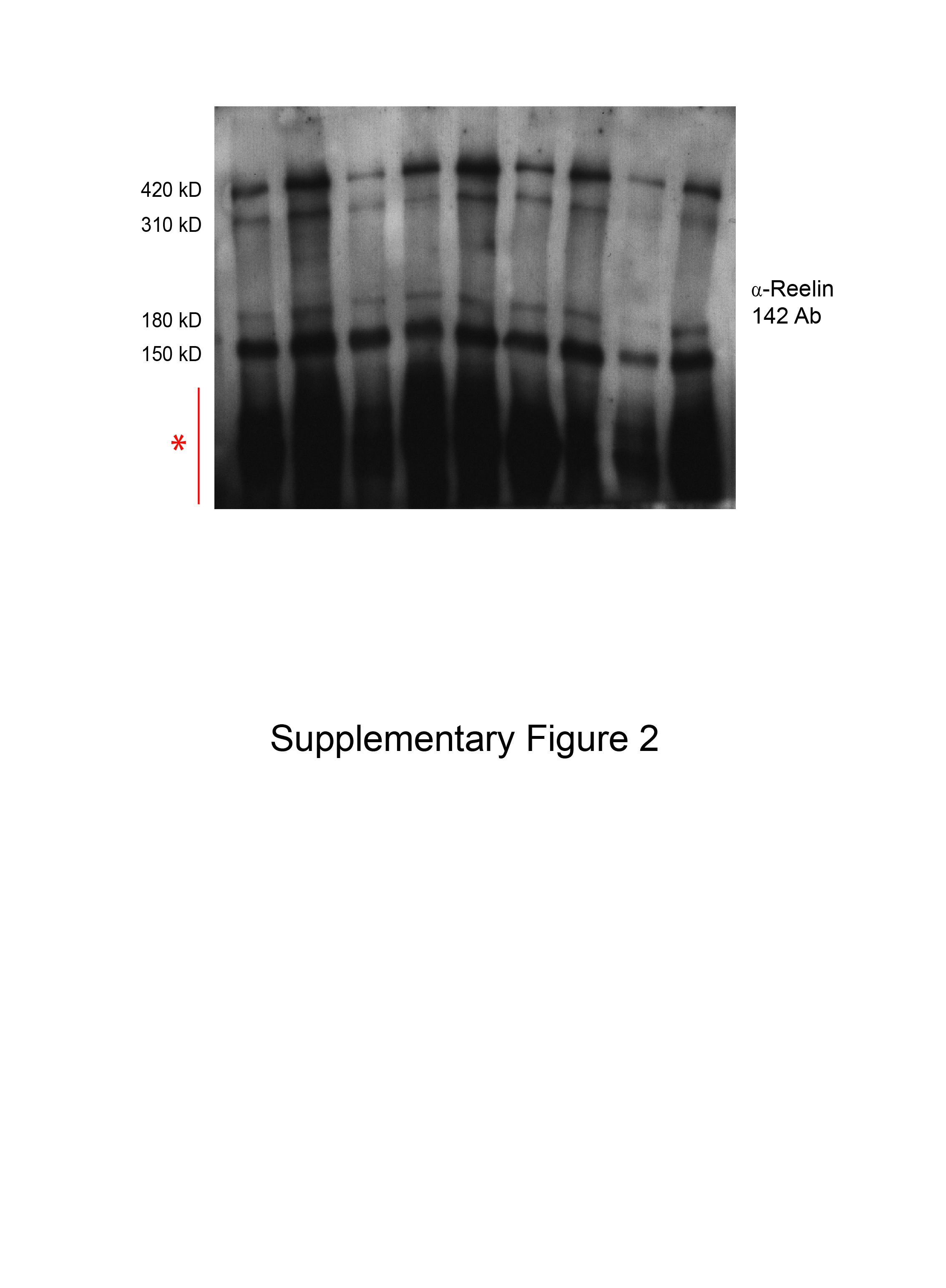

Supplement: Supplementary file 1 [file cells-09-01252-s001.zip › Supplementary Fig 2.jpg]

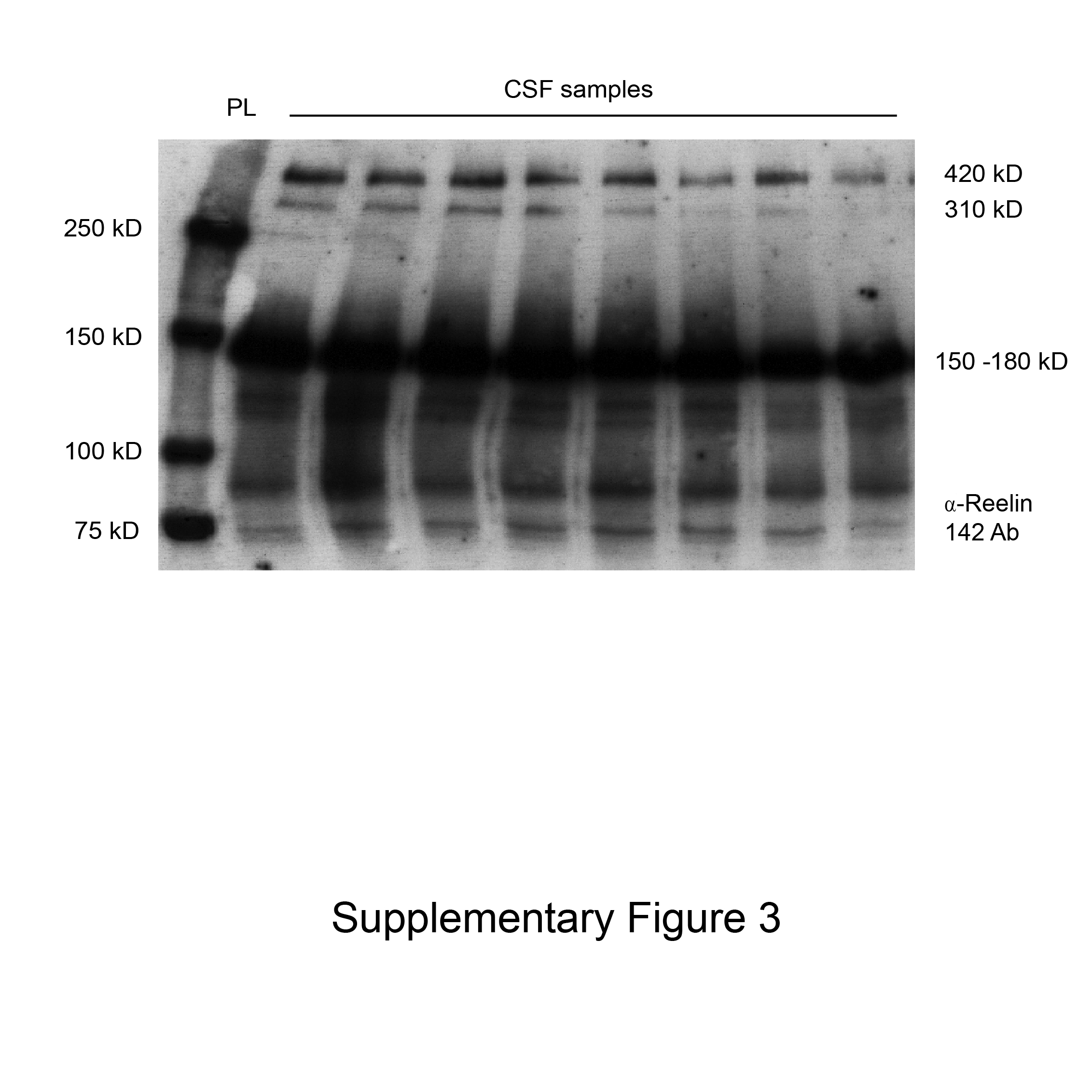

Supplement: Supplementary file 1 [file cells-09-01252-s001.zip › Supplementary Fig 3.jpg]
